# Prevalence and Significance of Antinuclear Antibodies in Biopsy-Proven Nonalcoholic Fatty Liver Disease: A Systematic Review and Meta-Analysis

**DOI:** 10.1155/2022/8446170

**Published:** 2022-08-12

**Authors:** Ling Luo, Qianqian Ma, Limin Lin, Hao Wang, Junzhao Ye, Bihui Zhong

**Affiliations:** ^1^Department of Gastroenterology of the First Affiliated Hospital, Sun Yat-sen University, No. 58 Zhongshan II Road, Yuexiu District, Guangzhou, China; ^2^Department of Infectious Diseases, Guangdong Provincial People's Hospital, Guangdong Academy of Medical Sciences, Guangzhou, China

## Abstract

**Aim:**

Associations between antinuclear antibodies (ANAs) and disease severity in nonalcoholic fatty liver disease (NAFLD) remain unclear. This study aimed to provide reliable estimates of ANA prevalence in subjects with biopsy-proven NAFLD and to investigate whether its associations with liver disease severity were established.

**Methods:**

Observational studies measuring ANA in NAFLD patients were derived from the PubMed, Embase, and Web of Science databases from inception to March 30, 2022. The effect size was presented as the pooled risk difference, unstandardized mean differences (MDs), and odds ratio (OR) with a 95% confidence interval (CI).

**Results:**

Thirteen articles involving 2331 patients were finally included. Among the subjects with biopsy-proven NAFLD, the overall prevalence of ANA positivity was high as 23% (95% CI: 19%-28%), but there were no statistically significant differences between ANA-positive and ANA-negative NAFLD patients in the levels of liver enzymes and blood lipids, grades of hepatocellular ballooning, lobular and portal inflammation, or risks of moderate-severe steatosis and significant fibrosis. However, the subgroup analysis showed that different geographic regions led to diverse results. ANA positivity was associated with a significantly elevated risk of significant fibrosis in the Eastern population (OR = 2.30, 95% CI: 1.30-4.06) but not in the Western population (OR = 1.00, 95% CI: 0.54-1.83).

**Conclusions:**

Serum ANA was present in approximately one-quarter of subjects with biopsy-proven NAFLD, but it conferred a greater risk of significant fibrosis only in Eastern but not Western populations.

## 1. Introduction

Nonalcoholic fatty liver disease (NAFLD), currently renamed metabolic-associated fatty liver disease (MAFLD), has become the leading cause of increasing chronic liver disease burden worldwide, with an estimated prevalence of 25% in the general population [1, 2]. NAFLD can be categorized histologically into two subtypes: the benign stage of simple nonalcoholic fatty liver (NAFL) and the progressive form of nonalcoholic steatohepatitis (NASH) with or without fibrosis, which is characterized by worsening metabolism and severe hepatic inflammation and hepatocyte injuries; the latter leads to an increased risk of death from all causes, including cardiovascular disease, diabetes, liver cirrhosis, and malignancies [3, 4]. However, the factors associated with liver lesion severity have still not been fully identified.

Antinuclear antibody (ANA) is a type of serum nonorgan-specific autoantibody and is induced by humoral immune responses against diverse recombinant nuclear antigens [5]. Seropositivity of ANA was relevant to autoimmunity but not specific to autoimmune disorders [6]. Elevated serum ANA titers might also be present in NAFLD without the context of autoimmune diseases or autoimmune hepatitis (AIH) [7]. For MAFLD-related management guidelines, high titers of ANA in patients with suspected liver fat infiltration would indicate that steatosis may be concurrent with or secondary to AIH, and further liver biopsy would be considered recommended for diagnosis [1, 3, 8–11]. Since the production of autoimmune antibodies, including ANA, was theoretically stimulated by several proinflammatory cytokines during humoral immune responses, there would be a possible biological impact of serum autoantibody positivity contributing to liver damage progression in NAFLD by activating hepatic macrophagocytes and stellate cells. Therefore, revealing the relationships among ANA and clinical aspects of NAFLD, especially metabolic abnormalities and histological characteristics, deserves further validation.

Previous reports investigated the occurrence of serum ANA positivity and found a prevalence ranging from 13% to 43% in NAFLD [12–14]. However, the results of these studies concerning whether ANA positivity correlated with liver histologic severity, including severe hepatic inflammation and steatosis or more advanced fibrosis, were inconsistent, possibly owing to relatively small-scale samples and different effects of age, sex, race, and disease severity. Moreover, whether ANA positivity is linked to metabolic abnormality patterns or whether these associations differ by geographic region remains unclear. Given the inconsistent conclusions, we conducted a systematic review and meta-analysis of observational studies to provide reliable estimates of the prevalence of ANA positivity and to investigate its associations with liver disease severity in subjects with biopsy-confirmed NAFLD.

## 2. Methods

This systematic review and meta-analysis was designed, performed, and reported in accordance with the proposal of reporting meta-analyses of observational studies in epidemiology (MOOSE) [15]. It has been registered in the PROSPERO registry (registration number: CRD42022302581).

### 2.1. Literature and Search Strategy

We searched eligible studies indexed in the PubMed, Embase, and Web of Science databases from the establishment of each database to March 30, 2022 (Supplementary Appendix 1). The search parameters included the keywords “nonalcoholic fatty liver disease” or “metabolic-associated fatty liver disease” or “metabolic dysfunction-associated fatty liver disease” or “nonalcoholic steatohepatitis” or “NAFLD” or “MAFLD” or “NASH” or “NAFL” or “fatty liver” or “liver steatosis” or “hepatic steatosis” combined with the terms “antinuclear antibody” or “autoantibody” or “ANA.” The reference lists of the included articles were also manually searched.

### 2.2. Eligibility Criteria and Study Selection

The inclusion criteria of the literature were as follows: (1) cross-sectional, case-control, or cohort studies; (2) studies that report serum ANA in NAFLD patients; and (3) the diagnosis of NAFLD based on the detection of hepatic steatosis by histological evaluation. Studies were excluded from this systematic review and meta-analysis according to the following criteria: (1) reviews, editorials, conference abstracts, case reports, unpublished articles, or studies on animals; (2) NAFLD patients coexisting with other liver diseases (e.g., alcoholic liver disease and viral hepatitis) or autoimmune disorders (e.g., systemic lupus erythematosus); and (3) studies in other languages that could not be translated into English.

### 2.3. Outcomes

The outcomes of this meta-analysis were as follows: (1) the prevalence of ANA positivity in NAFLD patients with a histological diagnosis. It was estimated by blood-based biomarkers irrespective of the threshold that was used to define ANA positivity. Different cutoff values (e.g., 1 : 40, 1 : 80, and 1 : 100) were attributed to different criteria and methods of serum ANA measurement [16–18]; (2) the difference of serum hepatic enzyme (alanine aminotransferase (ALT), aspartate aminotransferase (AST), *γ*-glutamyl transpeptidase (GGT), and alkaline phosphatase (ALP)), blood lipids (total cholesterol (CHOL) and triglycerides (TG)) between patients with ANA positive and negative; and (3) the difference of the histologic characteristics, including steatosis, hepatocyte ballooning, lobular inflammation, portal inflammation, and fibrosis. In the included studies, steatosis (graded 0-3), inflammation (0-3), and ballooning (0-2) were scored according to the NASH clinical research network system; fibrosis (0-4) was evaluated according to the METAVIR fibrosis scoring system [19, 20]. Currently, moderate-severe steatosis was defined as a steatosis score ≥2, and significant fibrosis was defined as a fibrosis stage ≥2 for the analyses.

### 2.4. Data Extraction and Quality Assessment

Two authors (L L and QQ M) independently selected and extracted data from eligible studies. For any inconsistencies in the extractions, corresponding authors (JZ Y and BH Z) participated in the discussion to achieve a final agreement. Using a standardized data extraction form, information on the following items was abstracted: first author, year of publication, country, race, study design, study group (children or adults), disease stage of the study group (NAFLD, NASH, or NAFL), diagnosis methods, number of ANA-positive cases, sample size, age, sex, body mass index (BMI), serum ALT level, quality score, and outcomes. In some studies, the prevalence of ANA positivity was not recorded directly but could be calculated from the available data. If important data could not be acquired directly from the article, we contacted the corresponding or first author by email to obtain primary reports; when they did not reply, the article was excluded. If data transformations were necessary during the analysis, we would also contact the authors for assistance; when original data were unavailable, we would apply the standard statistical formulas [21].

Article quality was evaluated by two researchers according to the assessment scale recommended by the Agency for Healthcare Research and Quality (AHRQ), which was suitable for cross-sectional/prevalence studies and encompassed 11 items with a maximum score of 11 points [22]. The quality of the literature was categorized as low quality (0-3), moderate quality (4-7), and high quality (8-11).

### 2.5. Statistical Analysis

In this study, data analysis was performed via the Software Review Manager Version 5.3 and Stata 12.0. We used the total number of ANA positivity among NAFLD subjects to estimate the pooled prevalence of ANA positivity. Then, subgroup analysis on the prevalence was conducted based on age (children and adults), sex (female and male), race (Asian and non-Asian), disease severity (NAFL and NASH), and geographic region (Eastern and Western) where these studies were performed. After data extraction and transformation, serum levels of hepatic enzymes and blood lipids, and grades of hepatocellular ballooning, lobular inflammation and portal inflammation were expressed as the means and standard deviation (SD); unstandardized mean differences (MDs) with 95% confidence intervals (CIs) were calculated to obtain the effect size between NAFLD patients who were ANA positive and negative. Additionally, the relationships between ANA and moderate-severe steatosis and significant fibrosis were assessed by the odds ratio (OR) with 95% CI. Notably, separate meta-analyses were performed if two or more studies were reported in the same group.

The potential for publication bias was assessed by inspection of funnel plot asymmetry. We further quantified the asymmetry using Egger's and Begg's tests; bias was considered significant for *P* values < 0.05. The heterogeneity across these studies was tested via *I*^2^ statistics, and an *I*^2^ value of greater than 50% indicated substantial heterogeneity. However, the value of the heterogeneity test was questionable if there were few included studies, so we used a random-effects model and further performed sensitivity analysis to verify the stability of the conclusions in this meta-analysis. In addition, publication bias was estimated by funnel plots, Egger's and Begg's tests.

## 3. Results

### 3.1. Characteristics of the Included Studies

**Figure 1:**
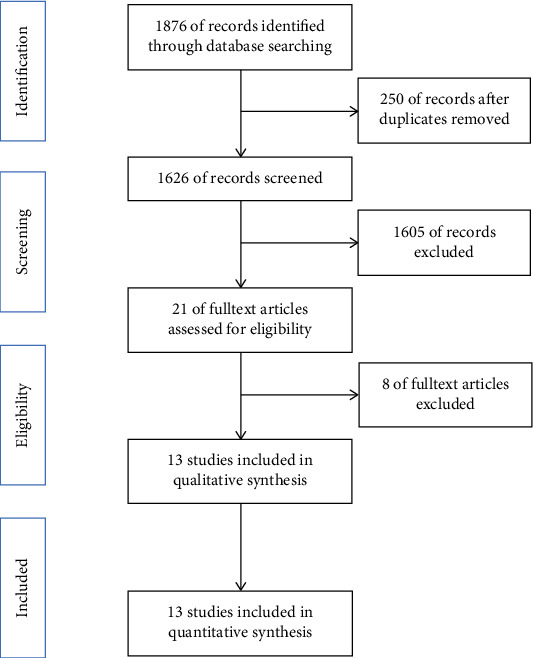
Flowchart of the study selection process.

A total of 1876 records were initially retrieved; 250 records were excluded because of duplicates being removed, and 1605 records were excluded based on title and abstract review; the remaining 21 articles were retrieved and assessed for eligibility for this meta-analysis by reviewing the full text. Of these full-text articles, 4 were excluded for not providing necessary data, 3 were excluded because NAFLD in those studies was not diagnosed by liver biopsy, and 1 was excluded because the authors adopted their own higher cutoff value to define ANA positivity. Thus, 13 articles consisting of 2331 patients with NAFLD were included in the final meta-analysis (Figure 1) [11–14, 23–31].  Table 1 outlines the detailed characteristics of the 13 included articles. Among them, three studies were performed in children, and the others were in adults; six studies were conducted in the United States (USA), three in Japan, one in China, one in Sweden, and the remaining two were multicenter studies that were performed in different countries. For article quality, 11 articles were considered to be of high quality, and two articles were considered moderate quality. No article was excluded because of low quality.

### 3.2. Meta-Analysis on the ANA Prevalence among NAFLD Patients

**Figure 2:**
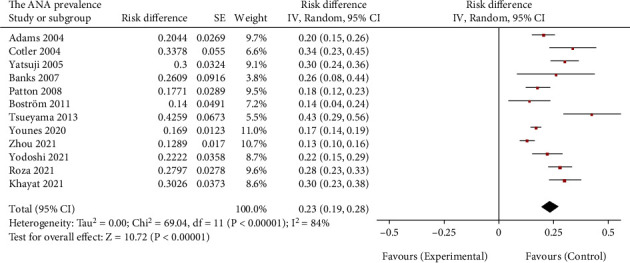
Overall prevalence of antinuclear antibody positivity in biopsy-proven NAFLD. ANA: antinuclear antibody.

As presented in Figure 2, the prevalence of ANA positivity was available in 12 studies consisting of 2260 participants with biopsy-confirmed NAFLD, in which the point prevalence of ANA positivity in patients ranged from 13% (95% CI 10 to 16%) (Zhou et al., 2021) to 43% (95% CI 29 to 56%) (Tsuneyama et al., 2013). Meta-analysis showed that the overall pooled prevalence of ANA positivity was 23% (95% CI 19 to 28%), with substantial heterogeneity (I^2^ = 84%). A further subgroup analysis showed that significant differences in the prevalence of ANA positivity were observed between the female and male subgroups (35% vs. 14%, *P* < 0.001) and between the NAFL and NASH subgroups (15% vs. 32%, *P* = 0.002). However, geographic region, age and ethnicity had no effect on the ANA prevalence (*P* = 0.48, 0.91 and 0.58, respectively) (Table 2).

### 3.3. Effects of ANA on Hepatic Enzyme and Blood Lipid Parameters

**Figure 3:**
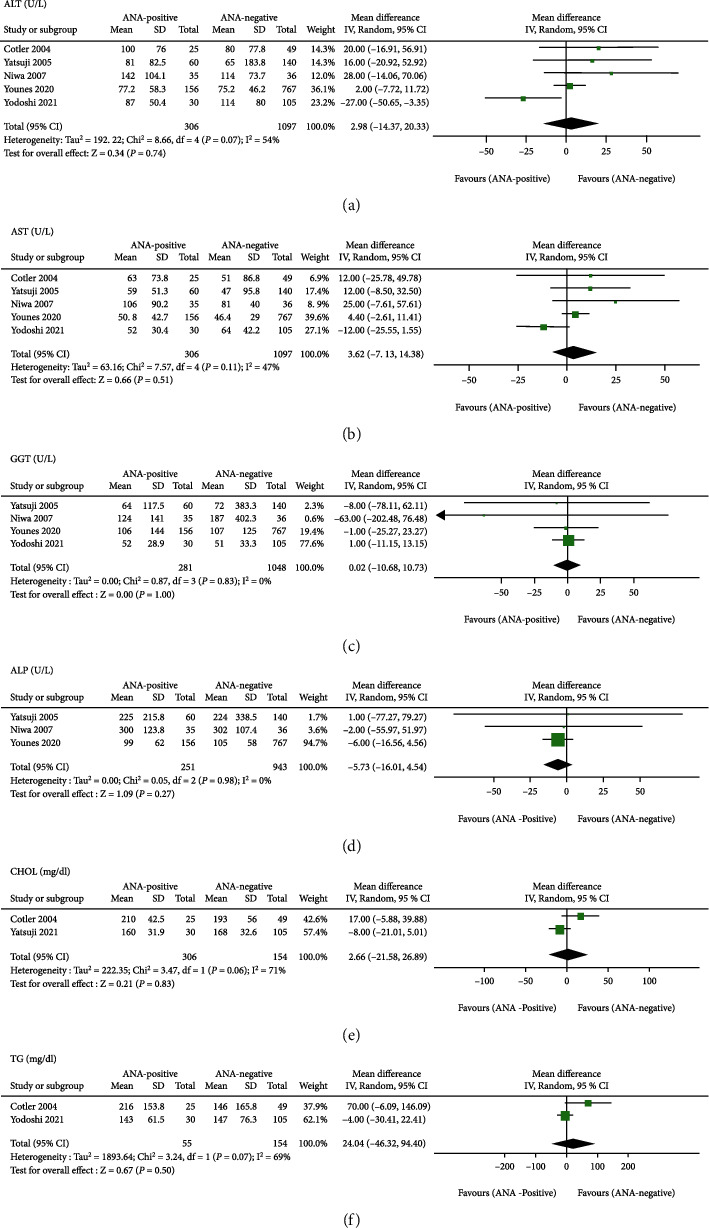
Meta-analysis of the effect of antinuclear antibodies on serum hepatic enzymes and lipid parameters of biopsy-proven NAFLD. (a) ALT (U/L). (b) AST (U/L). (c) GGT (U/L). (d) ALP (U/L). (e) CHOL (mg/dL). (f) TG (mg/dL). ALT: alanine aminotransferase; AST: aspartate aminotransferase; GGT: *γ*-glutamyl transpeptidase; ALP: alkaline phosphatase; CHOL: total cholesterol; TG: triglycerides.

**Figure 4:**
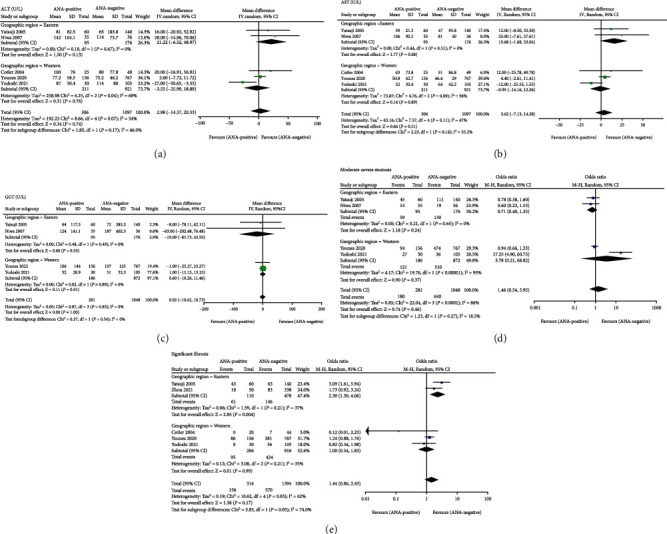
Subgroup analysis of the effect of antinuclear antibodies on NAFLD biochemical and histopathological indicators stratified by geographic region. (a) ALT (U/L). (b) AST (U/L). (c) GGT (U/L). (d) Moderate-severe steatosis (score of ≥2). (e) Significant fibrosis (score of ≥2). ALT: alanine aminotransferase; AST: aspartate aminotransferase; GGT: *γ*-glutamyl transpeptidase.

Liver enzymes are important biochemical parameters of NAFLD, so we assessed the association between ANA and these enzymes (Figure 3). Among the included studies, 5, 5, 4, and 3 studies compared the serum ALT, AST, ALP, and GGT levels between ANA-positive and ANA-negative NAFLD patients with a histological diagnosis, respectively, whereas the results of meta-analysis revealed that no significant differences in the four hepatic enzymes were found, with total random-effects MDs of 2.98 (95% CI − 14.37 to 20.33), 3.62 (95% CI − 7.13 to 14.38), 0.02 (95% CI − 10.68 to 10.73), and − 5.73 (95% CI − 16.01 to 4.54) U/L, respectively. Regarding blood lipids, only two reported data on CHOL and TG, but no statistically significant differences were noted when comparing the two lipids between NAFLD patients with ANA positivity and negativity in this meta-analysis (MD = 2.66 (mg/dl), 95% CI − 21.58 to 26.89 and MD = 24.04 (mg/dl), 95% CI − 46.32 to 94.40) (Figure 3). In the subgroup analysis by geographic region, the results of the two subgroups of ALT, AST, and GGT were consistent with the overall results (Figures 4(a)–4(c)).

### 3.4. Effects of ANA on Histological Characteristics

**Figure 5:**
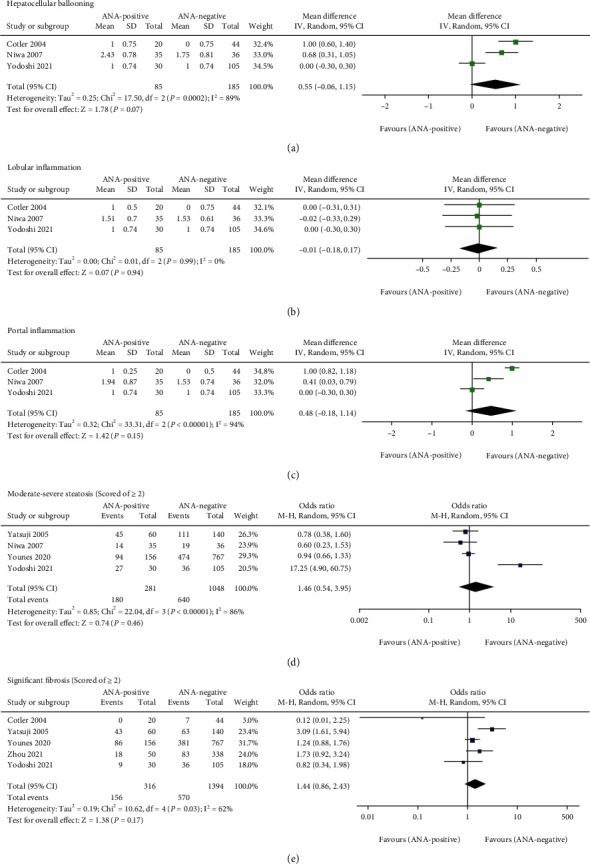
Meta-analysis of the effect of antinuclear antibodies on NAFLD histological characteristics. (a) Hepatocellular ballooning. (b) Lobular inflammation. (c) Portal inflammation. (d) Moderate-severe steatosis (score of ≥2). (e) Significant fibrosis (score of ≥2).

All of the included articles adopted liver biopsy to diagnose NAFLD, but only six studies compared the histological features between ANA-positive and ANA-negative patients. Out of these six studies, three studies provided enough data to calculate the means and SD for hepatocellular ballooning, lobular, and portal inflammation. As noted in Figure 5, the pooled random-effects MDs of ballooning, lobular and portal inflammation were 0.55 (95% CI − 0.06 to 1.15), − 0.01 (95% CI − 0.18 to 0.17), and 0.48 (95% CI − 0.18 to 1.14), respectively. Based on available data, this systematic review and meta-analysis further assessed the relationship of ANA with the risk of moderate-severe steatosis and significant fibrosis, which could be obtained in four and five studies, respectively. However, we found no difference in the risk of moderate-severe steatosis (scored of ≥2) and significant fibrosis (scored of ≥2) among NAFLD patients with ANA positivity compared with those with ANA negativity with the total ORs of 1.46 (95% CI 0.54 to 3.95) and 1.44 (95% CI 0.86 to 2.43), respectively (Figure 5). When subgroup analyses were conducted by geographic region, the two subgroups of the risk of moderate-severe steatosis were consistent with the overall results (Figure 4(d)). Interestingly, ANA-positive patients had a 2.30-fold (95% CI 1.30 to 4.06) higher risk of developing significant fibrosis than ANA-negative patients in the Eastern subgroup, while the Western subgroup did not reach a significant difference (OR = 1.00, 95% CI 0.54-1.83) (Figure 4(e)).

### 3.5. Publication Bias

Funnel plots of the ANA prevalence, liver enzymes, serum lipids, and histological characteristics were drawn to test for publication bias and are presented in Figure S1. The research points of overall prevalence, ALT, AST, CHOL, TG, hepatocellular ballooning, lobular and portal inflammation, steatosis, and fibrosis were basically symmetrical with little or no publication bias, while the funnel plots of GGT and ALP revealed that publication bias might exist. Furthermore, these were verified by Egger's test and Begg's test, showing that no publication bias was observed in all analyses except for ALP (*P* = 0.037 and 0.296, respectively) and hepatocyte ballooning (*P* = 0.027 and 0.296, respectively) (Table S1).

### 3.6. Sensitivity Analysis

Of the included studies reporting prevalence, none contributed to a 2% effect on the pooled ANA prevalence, suggesting that the result was stable and credible. Additionally, we calculated the prevalence by publication year. The estimated ANA prevalence was 25% (95% CI: 19% to 31%) in the studies conducted between 2000 and 2010 and 23% (95% CI: 17% to 28%) after 2010, indicating that the publication year did not significantly influence the results. Furthermore, we performed a sensitivity analysis to evaluate the robustness of the results of the meta-analysis of ANA on biochemical or histopathological indicators by omitting each individual study in turn and then calculating the total OR for the remaining studies. As shown in Figure S2, none of the studies significantly changed the conclusion in the analysis for liver enzymes, serum lipids, and histological features, suggesting that our statistics were relatively robust and reliable.

## 4. Discussion

To the best of our knowledge, this study is the first meta-analysis to report the prevalence of ANA positivity and the relationships of ANA with the disease severity in subjects with NAFLD diagnosed by liver biopsy. The present study found that almost one-quarter of biopsy-proven subjects were ANA-positive, but there were diverse associations between serum ANA and NAFLD in different regions. In the Western population, ANA was not related with NAFLD severity as determined by hepatic enzymes, serum lipids, and histological features, including steatosis, hepatocellular ballooning, lobular inflammation, portal inflammation, and fibrosis. However, NAFLD patients with ANA positivity had a higher risk of significant fibrosis compared with those with ANA negativity in the Eastern population.

Several published studies have investigated the prevalence of ANA in NAFLD patients. For example, a cross-sectional study performed in Japan involving 54 patients with biopsy-proven NASH reported that 42.6% of patients had positivity for serum ANA [29]. In addition, another multicenter study of 135 pediatric patients with NAFLD diagnosed by histological evaluation performed in the USA revealed that 30 patients (22.2%) were positive for serum ANA [11]. Recently, a study with 388 biopsy-confirmed NAFLD patients from China found that the prevalence of ANA positivity was 12.9% [14]. These discrepancies may be due to the various study populations with different races, ages, disease severity, and sex ratios. The present study comprehensively assessed all included published literature and showed that the overall prevalence of ANA positivity was 23%. Furthermore, our findings further showed that the ANA prevalence was higher in women than in men with NAFLD (35% vs. 14%), which showed a similar pattern of female predominance in autoimmune disorders. However, the underlying mechanism of sex differences remains unclear, and one plausible explanation was that sex hormones including estrogen, androgen, and prolactin balance played crucial roles in regulating immune and inflammatory responses, especially females which tended to be with higher serum levels of estrogen and prolactin (dramatic changes in status such as menopause, pregnancy, breastfeeding, and taking oral contraceptive pill) that are both proinflammatory hormones, thus promoting amplified T-cell/B cell overactivation and autoantibody production [32]. Gender differences in genetic factors (e.g., microchimerism, different influence of genes on sex chromosomes), as well as in lifestyle and environmental factors, including different occupational exposures to potential toxins, and frequencies of cigarette, cosmetics, and hair dyes use may be also involved in stimulating autoantibody formulation [33].

Serum ANA positivity was highly prevalent in patients with NAFLD, thereby raising the question of whether the presence of serum ANA was merely an epiphenomenon or directly associated with the occurrence and development of NAFLD. Thus, recently, a few researchers have investigated the relationship of ANA with NAFLD, but there have been conflicting results. An American study involving 225 NAFLD patients revealed that the simultaneous presence of two serum autoantibodies (ANA and anti-smooth muscle antibody) was associated with more severe histological damage, especially necroinflammation and liver fibrosis [23]. Moreover, Yodoshi et al. found that there was an association between ANA positivity and a greater degree of steatosis in a cohort of 135 children with a histologically proven diagnosis of NAFLD [11]. In contrast, a prospective multicenter study of 864 subjects with biopsy-confirmed NAFLD from the USA demonstrated that serum ANA was not related to more advanced histologic characteristics regardless of the positive cutoff titer of 1 : 160 or 1 : 40 among NAFLD patients [34]. Through a systematic review of all available articles, this meta-analysis showed a lack of association between ANA and hepatic enzymes, blood lipids, histological inflammation, and steatosis in NAFLD patients. However, with regard to liver fibrosis, Western and Eastern populations exhibit different relationships. The underlying mechanism of this phenomenon remains unclear, but these geographical variations raised a hypothesis that populations with different genetic backgrounds or various environmental exposures or both may have different types of autoimmune mechanism involved in the pathogenesis of NAFLD. This was supported by Zhou et al. that the association of serum autoantibody positivity and significant fibrosis was restricted to NAFLD patients carrying the PNPLA3 CG or GG genotypes [14].

More specifically, the current study demonstrated that there was no association between ANA and fibrosis stages in the Western population. It was consistent with a recent prospective cohort study in Western countries involving 923 NAFLD patients (156 ANA-positive patients) with a mean follow-up of 106 ± 50 months, which revealed that the long-term outcomes (e.g., end-stage cirrhosis, hepatic carcinoma, extrahepatic malignancy, and cardiovascular events) and survival of NAFLD patients were not affected by the presence of ANA [13]. Noteworthy, the presence of serum ANA was significantly associated with an increased risk of developing significant fibrosis in NAFLD patients in the Eastern population. In agreement with this finding, a previous study conducted in China also reported that serum autoantibodies positivity was correlated with more advanced stages of fibrosis in biopsy-confirmed NAFLD [14]. However, the biological mechanisms linking the presence of ANA and significant fibrosis in patients with NAFLD in the East are not fully understood. A possible explanation was that the production of autoantibodies in NAFLD may be a consequence of hepatic NKT cells accumulation, which has been reported to promote fibrosis in various liver diseases [35–37].

Notably, serum ANA positivity was also very common in subjects with AIH, in which one of the clinicopathological features was the presence of non-organ-specific autoantibodies [38]. The presence of serum ANA represented a challenge for clinicians not only in making a correct diagnosis but also in disease management. Therefore, our results did highlight that diagnostic and treatment strategies should be adapted relative to geographic regions. NAFLD patients with ANA positivity in Eastern but not Western countries should be closely monitored to provide early intervention before disease progression.

### 4.1. Study Strength and Limitations

One of the strengths was that the current study first investigated the overall prevalence and clinical significance of serum ANA via meta-analysis. Another advantage was that noninvasive methods (e.g., abdominal ultrasound) rather than histopathological evaluation have been ruled out. All of the included studies diagnosed NAFLD using liver biopsy, which is the gold standard and beneficial for making a credible conclusion.

However, certain limitations were also observed. First, although we have conducted a comprehensive and systematic search strategy, the number of studies included in this systemic review was limited, which might result in low statistical power. Hence, caution should be considered to interpret the pooled results. Second, several eligible articles were removed because they were unavailable for the necessary data, such as the levels of insulin or other metabolic markers. Third, there was statistical heterogeneity in this meta-analysis. The random-effects model could partially decrease but did not eliminate heterogeneity. Among studies providing the ANA prevalence, further subgroup meta-analyses were performed to explore the sources of between-study heterogeneity and revealed that proportion of women and NAFLD severity could partly account for heterogeneity. Among studies among studies reporting OR, a subgroup analysis stratified by Eastern and Western showed the potential effect of geographic regions on the risk of significant fibrosis. However, the possible influence of different regions on other important indicators (e.g., blood lipids and liver inflammation) could not be assessed due to the paucity of related data. Last but not least, the present study was a meta-analysis of observational studies, especially cross-sectional studies, thereby only showing an association instead of causality. More studies estimating the prognosis of ANA-positive NAFLD patients based on long-term follow-up are needed.

## 5. Conclusion

In summary, our meta-analysis demonstrated that serum ANA was present in approximately one-quarter of subjects with biopsy-proven NAFLD, but it was an important risk factor for developing liver fibrosis only in Eastern but not in Western populations. However, further investigations are warranted to confirm the reliability and generalizability of our findings and to elucidate the influence of geographical factors in the autoimmune mechanism of NAFLD.

## Figures and Tables

**Table 1:** Characteristics of the included studies.

Study	Country	Race	Study design	Study group	Diagnosis of NAFLD/NASH/NAFL	Diagnostic methods	Number of ANA-positive cases/total	Age, years	Sex (female/male)	BMI, kg/m^2^	ALT, U/L	AHRQ scale score
Adams 2004 [23]	USA	Caucasian	CS	Adult	NAFLD	Histology	46/225	46.0 ± 11.9	141/84	46.0 ± 11.9	94 ± 85	7
Cotler 2004 [12]	USA	Caucasian	CS	Adult	NASH	Histology	25/74	45 ± 13	44/30	NA	86.8 ± 77.2‡	8
Yatsuji 2005 [24]	Japan	Asian	CS	Adult	NAFLD	Histology	60/200	53 ± 20	100/100	NA	68 ± 184	10
Banks 2007 [25]	USA	Caucasian	CS	Adult	NAFLD	Histology	6/23	51.3 ± 5.0	13/10	32.8 ± 2.1	NA	10
Niwa 2007 [26] ^†^	Japan	Asian	CS	Adult	NASH	Histology	35/71	53.5 ± 15.9‡	46/25	NA	127.8 ± 90.0‡	9
Patton 2008 [27]	USA	Hispanic, Caucasian	CS	Children	NAFLD	Histology	31/175	12.4 ± 2.6	39/136	33 ± 5.2	89.8^‡^	9
Boström 2011 [28]	Sweden	Caucasian	CS	Adult	NAFLD	Histology	7/50	48 ± 10	13/37	NA	NA	7
Tsuneyama 2013 [29]	Japan	Asian	CS	Adult	NASH	Histology	23/54	54.8 ± 13.5‡	33/21	NA	102.1 ± 95.5‡	8
Younes 2020 [13]	Italy, UK	Caucasian	PC	Adult	NAFLD	Histology	156/923	47 ± 13	293/630	30.2 ± 5.8	75.5 ± 48.4	10
Zhou 2021 [14]	China	Asian	CS	Adult	NAFLD	Histology	50/388	40.6 ± 12.7‡	98/290	26.9 ± 3.3‡	58.7 ± 48.1‡	9
Yodoshi 2021 [11]	USA	Hispanic, Caucasian, Black, Asian	CS	Children	NAFLD	Histology	30/135	14 ± 4	54/81	NA	111 ± 76	11
Roza 2021 [30]	New Zealand, Singapore	Asian, European	PC	Adult	NASH	Histology	73/261	53 ± 12.9	126/135	30.61	112.3 ± 373.9	10
Khayat 2021 [31]	USA	Caucasian, Hispanic, Black	CS	Children	NAFL, NASH	Histology	46/152	12.2 ± 3.6‡	48/104	NA	NA	9

NAFLD: nonalcoholic fatty liver disease; NASH: nonalcoholic steatohepatitis; NAFL: simple nonalcoholic fatty liver; USA: the United States; UK: the United Kingdom; CS: cross-sectional study; PC: prospective cohort study; AHRQ: Agency for Healthcare Research and Quality; BMI: body mass index; ALT: alanine aminotransferase. Values are expressed as mean ± SD. ^†^ means this article did not report the prevalence of antinuclear antibody positivity but reported the liver related biochemical indexes and histology. ^‡^ means that the data was not recorded directly but could be calculated from the available data according to the following formulas: Total X = (X_1_∗N_1_ + X_2_∗N_2_)/(N_1_ + N_2_), Total SD = ((N_1_ − 1)∗(SD_1_)^2 + (N2 − 1)∗(SD2)^2)/(N1 + N2 − 2). In these formulas, *X*, *SD*, and *N* refer to the means, standard deviation, and sample size of each group; the subscript (e.g., 1 and 2) represents different group numbers.

**Table 2:** Results of meta-analysis regarding the prevalence of antinuclear antibody positivity in different conditions of biopsy-proven NAFLD.

Subgroup	Study (*n*)	Patient (*n*)	Prevalence (95% CI)	*I* ^2^	*P*
By age					0.91
Children	3	462	23% (16%-30%)	72%	
Adults	9	1798	24% (18%-29%)	86%	
By sex†					<0.001
Female	6	622	35% (25%-46%)	87%	
Male	6	1153	14% (11%-18%)	41%	
By race^‡^					0.58
Asian	3	642	28% (11%-44%)	94%	
Non-Asian	7	1622	23% (18%-27%)	60%	
By disease severity^§^					0.002
NAFL	2	67	15% (6%-23%)	0%	
NASH	5	574	31% (25%-37%)	49%	
By geographic region^#^					0.48
Eastern	3	642	28% (11%-44%)	94%	
Western	8	1757	22% (17%-26%)	68%	

NAFL: simple nonalcoholic fatty liver; NASH: nonalcoholic steatohepatitis; USA, the United States. ^†^ indicates that a total of 6 articles [11–14, 24, 29] reported the prevalence of antinuclear antibody positivity in men and women, and the other 6 articles were not reported, so a total of 6 articles were subgroup analyzed. ^‡^ indicates that 3 [14, 24, 29] and 7 [12, 13, 23, 25, 27, 28, 31] articles reported the prevalence of antinuclear antibody positivity in Asian and non-Asian populations, respectively, and the other 2 articles reported the prevalence in mixed populations inclusive of participants from both Asia and non-Asia, so a total of 10 articles were subgroup analyzed. ^§^ indicates that 2 [27, 31] and 5 [12, 27, 29–31] articles reported the prevalence of antinuclear antibody positivity in NAFL or NASH, respectively, and the other 7 articles were not reported, so a total of 5 articles were subgroup analyzed. ^#^ indicates that 3 [14, 24, 29] and 8 [11–13, 23, 25, 27, 28, 31] articles reported the prevalence of antinuclear antibody positivity in Eastern or Western countries, respectively, and the remaining study was conducted in both Eastern and Western countries, so a total of 11 articles were subgroup analyzed.

## Data Availability

Data sharing is not applicable to this article as no new data were created.

## Supplementary Materials

Supplementary Materials The following supporting information can be available in Supplementary materials. Appendix 1. Detailed Search Strategy; Table S1. *P* value for Egger's and Begg's tests for publication bias; Figure S1. Funnel plot of publication bias of the included trials. Figure S2. Sensitivity analysis chart included in the study.
