# Supplementary material for: Prevalence and Significance of Antinuclear Antibodies in Biopsy-Proven Nonalcoholic Fatty Liver Disease: A Systematic Review and Meta-Analysis
Source: Dis Markers. 2022 Aug 12;2022:8446170. doi: 10.1155/2022/8446170 (PMC9391168; doi:10.1155/2022/8446170)
Supplement: Supplementary Materials — The following supporting information can be available in Supplementary materials. Appendix 1. Detailed Search Strategy; Table S1. P value for Egger's and Begg's tests for publication bias; Figure S1. Funnel plot of publication bias of the included trials. Figure S2. Sensitivity analysis chart included in the study. [file 8446170.f1.doc]

**Supplementary**

**Appendix 1** Detailed Search Strategy

Pubmed n=363

("antibodies, antinuclear"[MeSH Terms] OR ("antibodies"[All Fields] AND "antinuclear"[All Fields]) OR "antinuclear antibodies"[All Fields] OR ("antinuclear"[All Fields] AND "antibody"[All Fields]) OR "antinuclear antibody"[All Fields] OR ("autoantibodies"[MeSH Terms] OR "autoantibodies"[All Fields] OR "autoantibody"[All Fields]) OR "ANA"[All Fields]) AND ("non alcoholic fatty liver disease"[MeSH Terms] OR ("non alcoholic"[All Fields] AND "fatty"[All Fields] AND "liver"[All Fields] AND "disease"[All Fields]) OR "non alcoholic fatty liver disease"[All Fields] OR ("nonalcoholic"[All Fields] AND "fatty"[All Fields] AND "liver"[All Fields] AND "disease"[All Fields]) OR "nonalcoholic fatty liver disease"[All Fields] OR ("metabolic-associated"[All Fields] AND ("fatty liver"[MeSH Terms] OR ("fatty"[All Fields] AND "liver"[All Fields]) OR "fatty liver"[All Fields]) AND ("disease"[MeSH Terms] OR "disease"[All Fields] OR "diseases"[All Fields] OR "disease s"[All Fields] OR "diseased"[All Fields])) OR (("metabolic"[All Fields] OR "metabolical"[All Fields] OR "metabolically"[All Fields] OR "metabolics"[All Fields] OR "metabolism"[MeSH Terms] OR "metabolism"[All Fields] OR "metabolisms"[All Fields] OR "metabolism"[MeSH Subheading] OR "metabolic networks and pathways"[MeSH Terms] OR ("metabolic"[All Fields] AND "networks"[All Fields] AND "pathways"[All Fields]) OR "metabolic networks and pathways"[All Fields] OR "metabolities"[All Fields] OR "metabolization"[All Fields] OR "metabolize"[All Fields] OR "metabolized"[All Fields] OR "metabolizer"[All Fields] OR "metabolizers"[All Fields] OR "metabolizes"[All Fields] OR "metabolizing"[All Fields]) AND "dysfunction-associated"[All Fields] AND ("fatty liver"[MeSH Terms] OR ("fatty"[All Fields] AND "liver"[All Fields]) OR "fatty liver"[All Fields]) AND ("disease"[MeSH Terms] OR "disease"[All Fields] OR "diseases"[All Fields] OR "disease s"[All Fields] OR "diseased"[All Fields])) OR ("non alcoholic fatty liver disease"[MeSH Terms] OR ("non alcoholic"[All Fields] AND "fatty"[All Fields] AND "liver"[All Fields] AND "disease"[All Fields]) OR "non alcoholic fatty liver disease"[All Fields] OR ("nonalcoholic"[All Fields] AND "steatohepatitis"[All Fields]) OR "nonalcoholic steatohepatitis"[All Fields]) OR ("naflds"[All Fields] OR "non alcoholic fatty liver disease"[MeSH Terms] OR ("non alcoholic"[All Fields] AND "fatty"[All Fields] AND "liver"[All Fields] AND "disease"[All Fields]) OR "non alcoholic fatty liver disease"[All Fields] OR "nafld"[All Fields]) OR "MAFLD"[All Fields] OR "NASH"[All Fields] OR "NAFL"[All Fields] OR ("fatty liver"[MeSH Terms] OR ("fatty"[All Fields] AND "liver"[All Fields]) OR "fatty liver"[All Fields]) OR ("fatty liver"[MeSH Terms] OR ("fatty"[All Fields] AND "liver"[All Fields]) OR "fatty liver"[All Fields] OR ("liver"[All Fields] AND "steatosis"[All Fields]) OR "liver steatosis"[All Fields]) OR ("fatty liver"[MeSH Terms] OR ("fatty"[All Fields] AND "liver"[All Fields]) OR "fatty liver"[All Fields] OR ("hepatic"[All Fields] AND "steatosis"[All Fields]) OR "hepatic steatosis"[All Fields]))

Embase n=926

('nonalcoholic fatty liver'/exp OR 'nonalcoholic fatty liver' OR 'metabolic fatty liver'/exp OR 'metabolic fatty liver' OR 'nonalcoholic steatohepatitis'/exp OR 'nonalcoholic steatohepatitis' OR (nonalcoholic AND ('steatohepatitis'/exp OR steatohepatitis)) OR 'fatty liver'/exp OR 'fatty liver') AND ('antinuclear antibody'/exp OR 'antinuclear antibody' OR (antinuclear AND ('antibody'/exp OR antibody)) OR 'autoantibody'/exp OR autoantibody)

Web of Science n=587

((TS=(nonalcoholic fatty liver disease)) OR TS=(metabolic-associated fatty liver disease)) OR TS=(metabolic dysfunction-associated fatty liver disease)) OR TS=(nonalcoholic steatohepatitis)) OR TS=(NAFLD)) OR TS=(MAFLD)) OR TS=(NASH)) OR TS=(NAFL)) OR TS=(fatty liver)) OR TS=(liver steatosis)) OR TS=(hepatic steatosis)) AND ((TS=(antinuclear antibody)) OR TS=(autoantibody)) OR TS=(ANA))

**Table S1.** *P* value for Egger’s and Begg’s tests for publication bias.

| **Characteristics** | **Study (n)** | ***P* for Egger’s test** | ***P* for Begg’s test** |
| --- | --- | --- | --- |
| ANA prevalence | 12 | 0.216 | 0.537 |
| Alanine aminotransferase | 5 | 0.683 | 0.221 |
| Aspartate aminotransferase | 5 | 0.639 | 0.806 |
| γ-glutamyl transpeptidase | 4 | 0.119 | 0.089 |
| Alkaline phosphatase | 3 | 0.037 | 0.296 |
| Total cholesterol | 2 | NR | 1.00 |
| Triglycerides | 2 | NR | 1.00 |
| Hepatocellular ballooning | 3 | 0.027 | 0.296 |
| Lobular inflammation | 3 | 0.999 | 1.00 |
| Portal inflammation | 3 | 0.385 | 1.00 |
| Moderate-severe steatosis | 4 | 0.503 | 0.734 |
| Significant fibrosis | 5 | 0.679 | 0.462 |

NR, not reported.

**Fig. S1** Funnel plot of publication bias of the included trials. a. Overall ANA prevalence (%); b. ALT (U/L); c. AST (U/L); d. GGT (U/L); e. ALP (U/L); f. CHOL (mg/dl); g. TG (mg/dl); h. Hepatocellular ballooning; i. Lobular inflammation; j. Portal inflammation; k. Steatosis ≥2 (%); l. Fibrosis ≥2 (%).

**g**

**h**

**i**

**e**

**f**

**a**

**d**


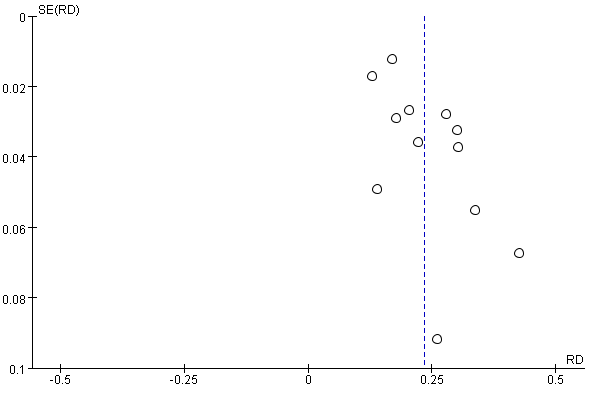

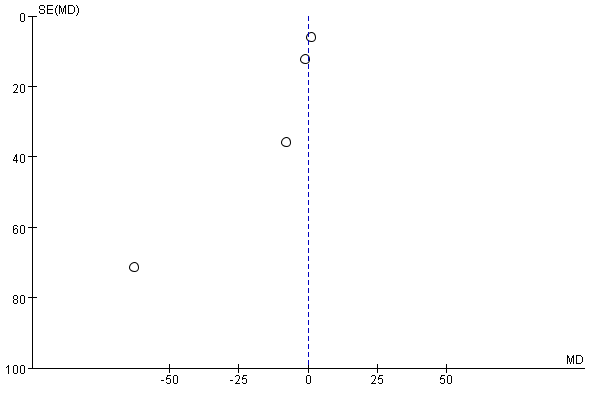

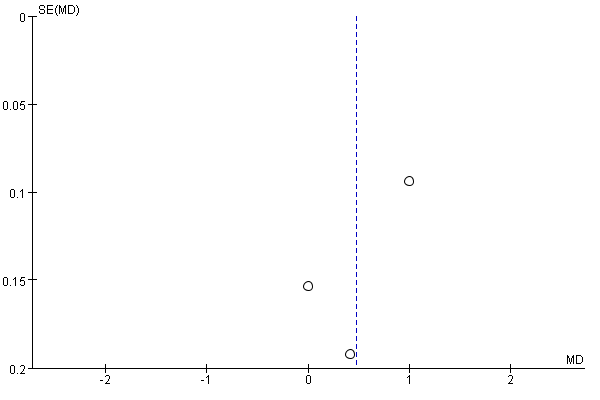

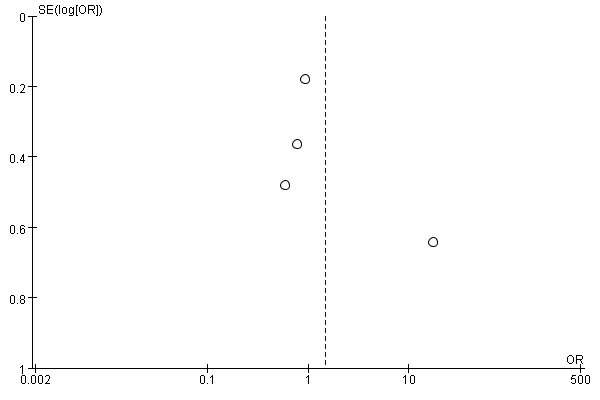

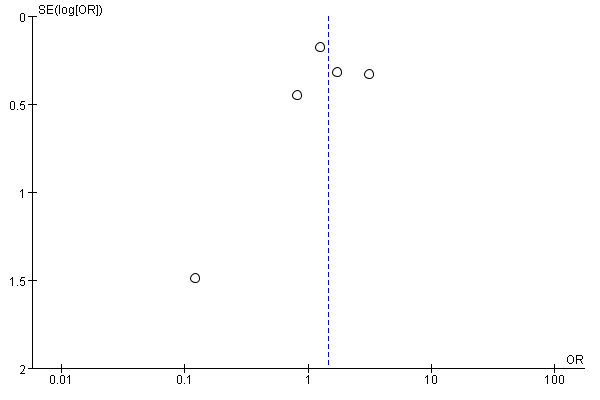


**TG (mg/dl)**

**Hepatocellular ballooning**

**Lobular inflammation**

**j**

**Portal inflammation**

**k**

**Steatosis ≥2 (%)**

**Fibrosis ≥2 (%)**

**l**

**Overall ANA prevalence (%)**

**ALT (U/L)**

**AST (U/L)**

**b**

**c**

**GGT (U/L)**

**ALP (U/L)**

**CHOL (mg/dl)**

)


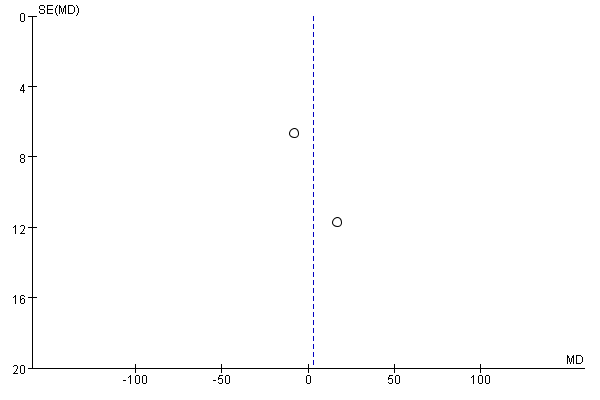

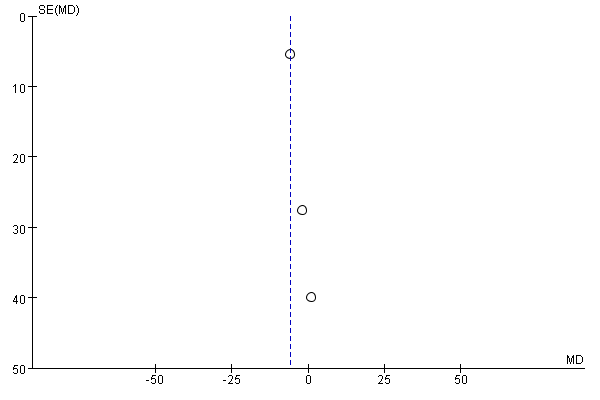

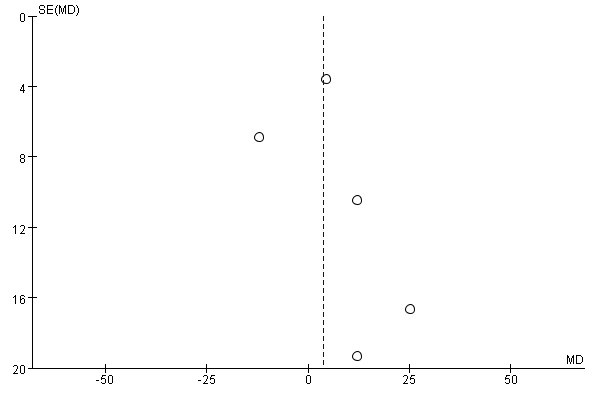

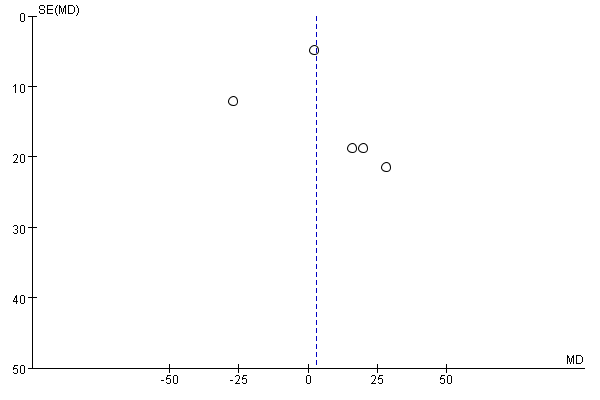

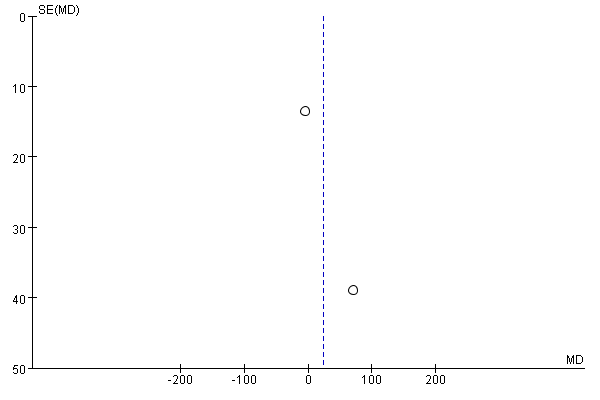

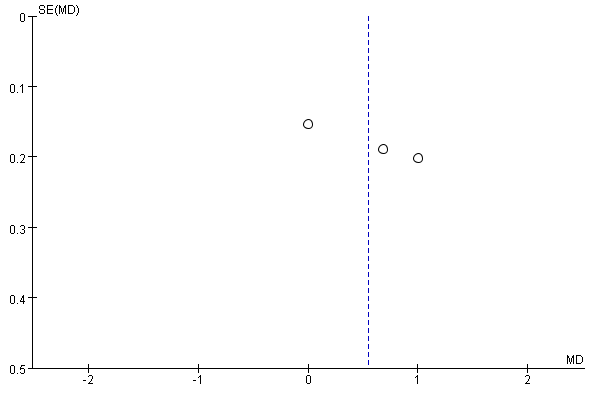

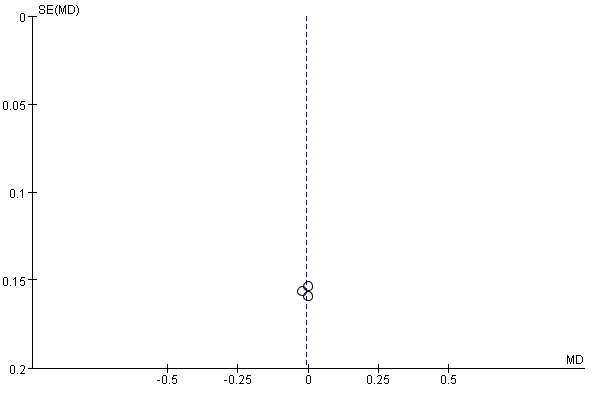


**Fig. S2** Sensitivity analysis chart included in the study. a. ALT (U/L); b. AST (U/L); c. GGT (U/L); d. ALP (U/L); e. CHOL (mg/dl); f. TG (mg/dl); g. Hepatocellular ballooning; h. Lobular inflammation; i. Portal inflammation; j. Steatosis ≥2 (%); k. Fibrosis ≥2 (%).

**ALT (U/L)**

**AST (U/L)**

**GGT (U/L)**

**a**

**b**

**c**

**ALP (U/L)**

**CHOL (mg/dl)**

**TG (mg/dl)**

**d**

**e**

**f**

**g**

**Hepatocellular ballooning**

**h**

**Lobular inflammation**

(%)

**i**

**Portal inflammation** (%)

**Steatosis ≥2 (%)**

**j**

**Fibrosis ≥2 (%)**

**k**
